# Are We Using Slow-Acting Symptomatic Chondroprotective Drugs Conscious Enough?

**DOI:** 10.2174/1874325001711010533

**Published:** 2017-05-31

**Authors:** Seyit Ali Gumustas, Kadir Oznam, Cagri Ata Mutlu, Yasin Emre Kaya, Ibrahim Yilmaz, Mehmet Isyar, Aliye Yıldırım Guzelant, Olcay Guler, Semih Akkaya, Mahir Mahirogullari

**Affiliations:** 1 Department of Orthopaedic and Traumatology, Dr.Lutfi Kirdar Kartal Training and Research Hospital, 34865, Istanbul,Turkey; 2 Department of Orthopaedic and Traumatology, Istanbul Medipol University School of Medicine, 34214Istanbul, Turkey; 3 Department of Medical Sciences, Acibadem Universitiy School of Medicine, 34752Istanbul, Turkey; 4 Department of Orthopaedic and Traumatology, Republic of Turkey, Ministry of Health, State Hospital, Corlu, 59850Tekirdag, Turkey.; 5 Department of Medical Pharmacology, Istanbul Medipol University School of Medicine, 34810Istanbul, Turkey; 6 Department of Orthopaedic and Traumatology, Acibadem Hospitals Group, Kadikoy, 34718Istanbul, Turkey.; 7 Department of Physical Medicine and Rehabilitation, Namik Kemal University School of Medicine, 59030Tekirdag, Turkey; 8 Department of Orthopaedic and Traumatology, Private Denizli Surgery Hospital, 20070Denizli, Turkey; 9 Department of Orthopaedic and Traumatology, Memorial Health Group, 34750Istanbul, Turkey

**Keywords:** Adverse effects, Cartilage, Chondroitin sulphate, Glucosamine sulphate, Hyaluronic acid, Symptomatic slow-acting chondroprotective drugs

## Abstract

**Background:**

Osteochondral injuries constitute an entity that is widespread and can be seen in patients of all ages. Actual treatment modalities aim to relieve pain, obtain full range of movement of the joint, and improve the quality of life. There are many slow-acting chondroprotective agents prevalently used in the United States that are classified as nutritional support but not as medicines . This study presents the importance of clinical adverse effect profiles as well as the pharmacological mechanism of action and application of combinations of drugs that are widely prescribed and not subjected to control.

**Methods:**

Electronic databases were searched with keywords about the chondroprotective drugs without any language restriction. Evaluations of the descriptive statistics were represented *via* Microsoft Office Excel 2010 lists in the form of a mean±standard deviation or frequency (%). The first evaluation showed that 1502 studies were potentially relevant. Following exclusion of the 1277 studies which were not clinical, full versions of the remaining 225 studies were subjected to further evaluation. No controlled, blinded, randomized and/or comparative studies met the inclusion criteria of the study, and no studies evaluated the comparative clinical results of the hyaluronan of different molecular weights.

**Results:**

The findings of this study concluded that especially when prescribing drugs with ingredients like GS and CS, many patients’ pre-existing conditions must be considered, such as whether the patient has a glucose intolerance or not. Additionally, mineral toxication should be considered since the drugs contain minerals, and after the application of injected hyaluronan, complications should be considered.

**Conclusion:**

Clinical, controlled and comparative studies about the use of chondroprotective drugs must be performed to define the benefits of these drugs, if any, in order to determine the most suitable time for operative intervention.

## INTRODUCTION

Osteochondral damage may develop in articulations in all life cycles due to congenital, traumatic, vascular, degenerative, infective, inflammatory and metabolic diseases [1, 2]. Many experimental studies exist for treatments to repair osteochondral damage.

 These treatments not only aim to make patients capable of accomplishing daily tasks, but also to improve patient’s quality of life during early term. While choosing appropriate treatment methods, specific patient information must be considered to choose the most effective treatment method with the lowest cost [1-6].

Most preferred treatments focus on controlling pain, eliminating movement restriction and increasing quality of life. To this end, analgesics, non-steroid anti-inflammatory drugs, intraarticular (IA) injections that do not contain steroids and physical and rehabilitation methods are most common. Moreover, the use of pharmacologic agents applied as viscosupplementation that are symptomatic and slow-acting, such as autologous platelet-rich plasma and/or hyaluronic acid (HA), have been increasing [7-10].

Condroprotective agents are pharmaceutical products that have not been classified as drugs in many countries, including the United States. They have been classified as food supplements and have not been tested by many institutions, except the *American Food and Drug Administration.* Notwithstanding, they are frequently prescribed for patients by doctors with the purpose of relieving the pain.

Patients who take food supplements and/or modifying osteoarthritis drugs without doctor’s advice may experience various undesirable effects frequently seen in drug-drug and drug-nutrition interaction, due to unknown and uninvestigated side effects of the drugs. As long as these drugs cannot be prescribed by clinicians, evaluating positive and/or negative side effects is not possible. As research regarding condroprotective drugs is limited and the undesired effect profile is not complete, doctors must use cost-effective treatments that can result in morbidity or mortality and other unforeseen results.

In the present systematic review, studies regarding symptomatic slow-acting chondroprotective drugs which are widely used yet uncontrolled were investigated. The study aimed to consider pharmacologic effect mechanism and application combinations, emphasizing side effect and/or adverse effect profiles that may occur after patients use chondroprotective without any prescription from a doctor.

## MATERIALS AND METHODS

### Search Strategy

**Fig. (1):**
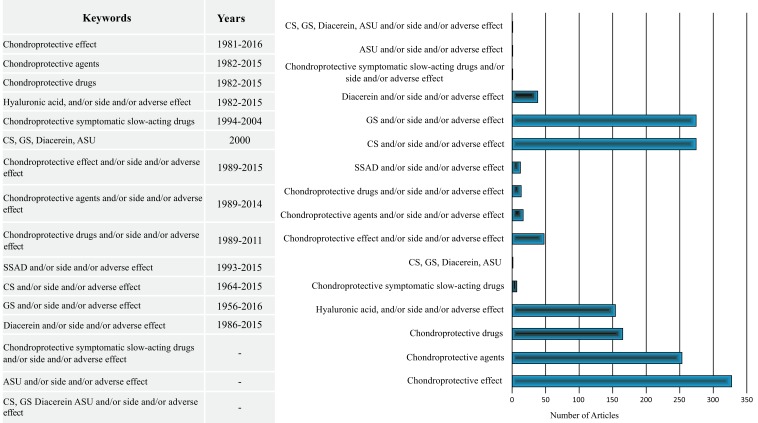
Scanning Process. The symbolizes are as follow: CS: chondroitin sulfate; GS: glucosamine sulfate; SSAD: symptomatic slow-acting drugs; HA: hyaluronic acid; ASU: avocado and soya unsaponifiables.

Electronic databases such as US National Library of Medicine National Institutes of Health (NLM) (PubMed), Embase, OVID and Cochrane Library were searched from the year 1956 to January 22, 2016 using keywords such as “chondroprotective effect,” “chondroprotective agents,” “chondroprotective drugs,” “chondroprotective symptomatic slow-acting drugs,” “chondroitin sulphate (CS),” “glucosamine sulphate (GS),” “diacerein,” “avocado and soya unsaponifiables (ASU)” and “hyaluronic acid (HA),” along with “side effect” or “adverse effect” (Fig. **1**).

Studies by Lijmer *et al.* [11, 12] were used to determine the proof level of the studies. All bibliographies were also reviewed and reference lists were re-evaluated to find appropriate articles. Unpublished works were not included in the study. Comments, letters, editorials, protocols, guides, meta analyses and compilation works were excluded. Most cited studies were found in *Web of Science* and *Scopus*. All references and citations were examined to avoid repetition.

### Eligible Criteria

The studies which were not clinical, controlled, blind and non-comparative concerning CS, GS, diacerein, ASU, and HA were not included.

### Data Collection and Evaluation

References were selected independently by all authors. Selection bias from potential masking was extensively monitored. With the aim of providing verification, all references were examined by each author. In the cases when at least two authors did not reach a consensus, the senior author examined the case(s).

### Statistical Evaluation

The evaluation of data and descriptive statistics were processed using Microsoft Excel lists (Microsoft Office 2010 program) as mean±standard deviation or frequency (%).

## RESULTS

After the first evaluation of sources, 1502 studies were found to be potentially related. Further, 1277 nonclinical studies were excluded, and 225 articles were excluded because they did not match the inclusion criteria (controlled, blind, randomized and/or comparative).

## DISCUSSION

Treatment expenses for osteoarthrosis patients are high [13]. Current treatment methods to avoid osteoarthrosis damage are growth factors, anticytokines, anti-inflammatory drugs and stem cell-based treatment strategies. Among these treatments, stem cell-based treatments place the greatest burden on the health economy [14]. Research shows that cartilage tissue similar to hyaline cannot be obtained [15]. Independent studies carried out in small sampling and research groups due to economic and time constraints regarding GS, CS, HA, ASU, symptomatic slow-acting chondroprotective drugs, and diacerein pharmacologic treatment protocols have been increasing. Because there are so many differences emerging in the available research, researchers have begun to work together when gathering data [16].

The present systematic review aims to uncover pharmacologic effect mechanisms, clarify side/adverse effect profiles and evaluate clinical importance of symptomatic slow-acting chondroprotective drugs.

Data obtained from different resources will be transformed into the same effect size. Then, the effect size of the studies included in the analysis is calculated [17]. In addition, this study tests whether effect size is distributed homogeneously. As a result, if effect size shows a homogeneous distribution, the fixed effect model should be used; otherwise, the random effect model should be used [17-20].

There were few to no clinical studies found meeting inclusion criterion with high proof value. For this very reason, effect size or homogeneity tests were not performed. This is the limitation of this study. However, the present study still encompasses the first studies where CS, GS, diacerein, and ASU were evaluated together along with side/adverse effects.

Research shows that HA, which was isolated from swine vitreous humor, has positive biochemical effects on cartilage cells, and its half-life in cartilage tissue was 2-3 weeks [21, 22]. HA, or hyaluronan, is the only linear polysaccharide which does not contain a sulfate group among glucuronic acid groups as a result of glycosidicality [22].

HA does not have drug-nutrition interaction, and it does not cause adverse reactions except in cases involving chicken allergies [21]. HA molecule weight is 4-5 dalton in an ordinary joint. The average hyaluronan amount is 4-8 mg in joints and 0,35gr/100ml concentration in joint liquid [23].

It is mentioned that erosive effect could be limited in joints by means of HA treatment [24]. HA is recommended for early cases when NAID is contraindicated, not tolerated, and corticosteroids are ineffective [25].

In the limited studies concerning osteoarthritis viscosupplementation treatment, it is indicated that HA has healing effects in dry joints due to its its lubricant characteristic [26]. It is reported that patients protected by medium grade morphological changes and who have joint spacing respond to the treatment better, and the results of advanced osteoarthritis cases are more limited. HA is effective in knee osteotrite, which is resistant to conventional treatments, but is promising in other joint applications. It is reported that HA has long standing positive effects despite its half-life. Long lasting effects result from recovery of lubricity and elasticity with its chemical structure, reconstruction of “*viscosupplementation*” or joint rheology, observation of anti-inflammatory and antinociceptive effects, normalization of endogeny HA synthesis and protection of cartilage such as “biosupplementation” characteristics [26-30].

In a randomized study in which there were 4866 patients, HA was evaluated between the fourth and 13^th^ weeks as well as the 14^th^ and 26^th^ weeks concerning knee pain and functionality. When cases were compared with the values before HA injection, improvements in knee pain and functionality were observed [10].

In studies regarding intraarticular HA application, it is reported that HA does not outclass corticosteroid applications and may be effective on regeneration of chondrocyte along with its analgesic features. In addition to contradictory results, it is indicated that IA HA injections, which are repeatedly applied, do not affect the OA process or not slow down. After injections, intrapelvic abscess and septic arthritis cases were discovered [31].

It has been proven through an *in-vitro* clinical studiy that biological activity is better in terms of high molecular HA preparates in-vitro environments, as high molecular weight showed a better performance [31, 32]. Preference of high molecular HA to low molecular HA is controversial.

Diacerein is in 4,5-diacetoxy-9,10-diokso-anthracene-2-carboxylic acid structure as a chemical called diacetilrein. This pharmaceutic agent plays a role in destruction of the cartilage, synovial inflammation and transformation of the subchondral bone. Diacetilrein is an inhibitor of interleukin beta (IL-1β), which could trigger the production of many pre or pro-inflammatory factors, including cytokines, cyclooxygenasei, prostaglandins, nitric oxide and matrix metalloproteinases [33, 34].

Diacerein, which is taken orally with systemic action, arrives at hepatic passing, and is diacetyled to rhein and absorbed before it circulates. After 100 mg Diacerein is taken as a single dose, free rhein plasma value was only 8-10 g/ml [34-36].

Diacetilrein is not appropriate for those who have hypersensitivity to Diacerein, anthraquinone derivatives and excipients, or for those with hepatic deficiency, inflammatory bowel disease and intestinal obstruction. Furthermore, its use is contraindicated for those under 18 years old along with those who are pregnant or breastfeeding. Antacids obtained from magnesium, aluminum and calcium may reduce absorption of diacerein from digestive system. Therefore, there should be at least two-hours-time interval between the use of diacerein and preparates containing diacerein. It was observed that when diacerein is used concomitant with warefarin, phenytoin, indomethacin, paracetamol, salicylic acid, glibenclamide, hydrochlorothiazide, cimetidine and anti-inflammatory drugs that are not in steroid structure, there was no pharmacologic interaction. However, in 2014, the European Medicines Agency reported that diacerein causes severe diarrhea [37].

In studies which were carried out in-vitro, diacerein was shown to stimulate cartilage growth factors, such as transformer growth factor-beta1 (TGF-β1), even in the presence of IL-1β, as well as to stimulate the syntheses of components of cartilage matrix, such as glycosaminoglycan and HA [38, 39].

It is reported that GS, *2-amino-2-deoxy-beta-D-glucopyranose*, and CS, *2-amino-2-deoksi-D-glukoz sulphate*, contain a wave sugar chain in *N-acetylgalactosamine* and *glucuronic acid* structure [40]. In pharmacokinetics studies involving human and experimental living mammals, it was reported that single dose 300 mg CS was absorbed by first degree kinetics after being taken orally. It was also emphasized that when it is applied in OA cases as 800 mg multiple doses, its kinetics did not change and the effect was the same [41].

In a study about the reliability and clinical activity of GS and CS in the cases with knee OA, it was found that when GS and CS were used alone or in combination, they were as effective as celecoxib and placebo groups [42].

Additionally, CS, which does not interact with liver cytochrome p450 enzyme, does not have medication-medication interaction and can be used together with the drugs used in the treatment of hypertension, hyperlipidemia and diabetes [43, 44].

It is reported that there are effect mechanisms that are not very clear in the way that ASU stimulates TGF-β1 and may stimulate matrix synthesis of chondrocyte due to reduction of matrix metalloproteinase production. In chondrocytes, production of the extracellular matrix is stimulated, such as type II collagen and proteoglycans. Although a study indicates that chondrocytes increase TGF- β1 and TGF- β2 levels in knee joint in dogs, its effect on full-thickness articular cartilage lesions is not revealed in clinics [45].

. Maheu et al report that joint spacing progress decelerated in a controlled study in which they evaluated 399 cases with symptomatic hip OA for three years [46].

When treatment-resistant OA cases are older and multiple drugs have been used, treatment possibility becomes more difficult. As a result of this, these cases lean towards alternative treatment methods.

Absorption speed and medication rates in older cases may differ. In older cases where the absorption rate slows down, the total body water and lean body weight decrease, and body fat increases. For this very reason, while the volume of distribution in medications with high diluent in fat increases, volume of distribution of hydrophilic medications decreases. Also, it is observed that total plasma protein does not change and albumin fraction decreases in older cases. Due to decreased albumin concentration, free medication levels in plasma increase when medications that affect binding proteins are used in older cases. Moreover, they compete with each other in order to bind protein due to multiple medications, which increases the risk of side effect presence [47-49].

Additionally, older cases also experience changes in the number and affinity of receptors, secondary message systems and cell response. Chronic illnesses that increase as people get older augment medication is needed, leading to side/adverse effects, drug-drug interaction and drug-nutrition risks [47-49].

As a result, because these drugs may contain inactive ingredients such as lactose, it may not be safe to prescribe these medications to patients with lactose intolerance.

In drugs, in which GS and CS are combined, there is low level of manganese. In patients who consume cereals, rice, soya bean, egg, hazelnut, olive oil, green bean and oysters, there may be an accumulation of drugs in the liver, kidney, pancreas, endocrine glands, respiratory tract and brain after manganese absorption. Manganese is toxic in high concentrations.

Due to manganese accumulation, muscle weakness, headache and insomnia may occur in addition to the increase in psychotic illnesses and malignites.

One of the limitations of this research is publication bias as a result of reviewed studies. Another limitation is that the studies available for review contain data obtained from previous studies. When the studies under review were performed earlier, it is not possible to make an amendment for them.

As a result, in this study, 225 articles were not found to be inclusive, as clinical investigations were not carried out where molecular weight of hyaluronic acid was compared.

There wasn’t a study regarding long term follow-up results after the use of these prescribed pharmaceuticals. Additionally, many patients in previous studies were prescribed without considering whether they were diabetic or glucose intolerant. It was also discovered that toxication due to the accumulation of manganese was not considered. Previous studies lack discussion or evidence regarding postponement of treatment or needlessness of surgery after the use of slow-acting symptomatic chondroprotective drugs.

## CONCLUSION

Some further studies will be performed where long term results of slow-acting/symptomatic chondroprotective are considered and side and/or adverse effects are compared. Afterwards, exact information is to be put forward regarding conservative and surgical treatment timing.
